# The In Vitro Effects of Romina Strawberry Extract on 3D Uterine Leiomyosarcoma Cells

**DOI:** 10.3390/nu15112557

**Published:** 2023-05-30

**Authors:** Stefania Greco, Pamela Pellegrino, Francesca Giampieri, Franco Capocasa, Giovanni Delli Carpini, Maurizio Battino, Bruno Mezzetti, Stefano Raffaele Giannubilo, Andrea Ciavattini, Pasquapina Ciarmela

**Affiliations:** 1 Department of Experimental and Clinical Medicine, Università Politecnica delle Marche, 60126 Ancona, Italy; s.greco@staff.univpm.it (S.G.); p.pellegrino@pm.univpm.it (P.P.); 2 Research Group on Food, Nutritional Biochemistry and Health, Universidad Europea del Atlántico, 39011 Santander, Spain; f.giampieri@staff.univpm.it (F.G.); m.a.battino@staff.univpm.it (M.B.); 3 Department of Agricultural, Food and Environmental Sciences, Università Politecnica delle Marche, 60100 Ancona, Italy; f.capocasa@staff.univpm.it (F.C.); b.mezzetti@staff.univpm.it (B.M.); 4 Department of Clinical Sciences, Università Politecnica delle Marche, 60126 Ancona, Italy; g.dellicarpini@staff.univpm.it (G.D.C.); s.r.giannubilo@staff.univpm.it (S.R.G.); a.ciavattini@univpm.it (A.C.); 5 International Research Center for Food Nutrition and Safety, Jiangsu University, Zhenjiang 212013, China

**Keywords:** leiomyosarcoma, phytochemicals, strawberry, 3D culture

## Abstract

Leiomyosarcoma is an aggressive soft tissue sarcoma derived from the smooth muscle cells of the uterus. We tested the effect of Romina strawberry extract treatment on three-dimensional cultured uterine leiomyosarcoma cells. We established 3D cultures in agarose gel, where the cells seeded were able to form spheroids. We performed the observation and counting of the spheroids with a phase-contrast optical microscope, finding a decrease in the number of spheroids formed in the plates after 24 and 48 h treatment with 250 µg/mL of cultivar Romina strawberry extract. We also characterized the spheroids morphology by DNA binding fluorescent-stain observation, hematoxylin and eosin stain, and Masson’s trichrome stain. Finally, the real-time PCR showed a reduced expression of extracellular matrix genes after strawberry treatment. Overall, our data suggest that the fruit extract of this strawberry cultivar may be a useful therapeutic adjuvant for the management of uterine leiomyosarcoma.

## 1. Introduction

The most frequent gynecologic neoplasms are smooth-muscle tumors [1,2]. From the histological point of view, in most cases they present benign aspects, being characterized by the absence of cytologic atypia or necrosis as well as by low mitotic index; these neoplasms are usually classified as leiomyoma [3]. However, in case of moderate cytologic atypia and/or mitotic index > 10/10 high power fields and/or coagulative tumor cell necrosis, a diagnosis of malignancy can be made if at least two of the previous features are concomitantly present according to Stanford criteria; these types of tumors are usually identified as leiomyosarcoma.

Uterine leiomyosarcoma is a rare malignancy whose etiologic origin is still unknown and accounts for approximately 1–2% of all malignant tumors of the uterus and for 60–70% of all uterine sarcomas [3]. In the United States, the incidence is estimated at around six cases per one million women with a median age at diagnosis of 51 years. Histologically, it is a soft-tissue sarcoma that arises from the smooth-muscle cells of the myometrium, representing the malignant counterpart of uterine leiomyoma and is characterized by abnormal, uncontrolled cellular growth that invades and can make metastases [4]. It can be classified into epithelioid and myxoid and is recognized and diagnosed only and exclusively on a histological basis. Hypercellularity, severe nuclear atypia, necrosis, and high mitotic rate (greater than 15 mitotic figures for 10 high-power fields) are the main common traits [5,6].

Since currently there are no efficient diagnostic biomarkers [7], the diagnosis is often controversial, also because it is not simple to predict tumor behavior on the basis of differentiation or histologic patterns as well as it being difficult to discriminate between uterine leiomyosarcoma and other forms of uterine smooth-muscle tumors. Although the usual type of leiomyoma is the most frequent, different leiomyoma variants exist, such as apoplectic leiomyoma, lipoleiomyoma, cellular leiomyoma, and bizarre or atypical leiomyoma. In some cases, especially for bizarre leiomyoma, is difficult to discriminate between benign and malignant lesions. For this reason, the 2020 World Health Organization (WHO) classified the smooth muscle neoplasms in leiomyoma, leiomyosarcoma (LMS) and introduced the category of smooth-muscle tumors of uncertain malignant potential (STUMP) [8]. Moreover, even if the survival rate reported at 5 years for this type of tumor is variable, there is a high risk of recurrence because of its extremely aggressive features, which unfortunately makes the prognosis generally poor [3,9]. Additionally, since little is known about its aetiology and pathophysiology, a specific strategy for adequate therapy is not still known, consisting mostly of surgical resection, radio, and/or chemotherapy [10].

Historically, natural compounds have exerted a crucial role in the discovery and development of new drugs or therapies, particularly for treating common human pathologies, including cardiovascular diseases and some types of cancer [11]. Fortunately, over the years, great interest has been renewed in the use of these natural compounds, as demonstrated by modern pharmaceutical industries that use more frequently dietary phytochemicals as a source of potential new drugs for many types of tumors with well-known antitumor effects [12,13]. 

In fact, many dietary phytochemicals, found especially in plant foods, such as fruits, vegetables, cereals, legumes, herbs, spices, nuts, and seeds, are able to exert tumor preventive and/or tumor-destroying properties by promoting antiproliferative effects. Their ability to modulate tumorigenic processes, such as apoptosis, proliferation, fibrosis, cell metabolism, and angiogenesis among others, has made them to be an alternative option for different diseases [14]. Therefore, dietary bioactive compounds could be effective against uterine leiomyosarcoma as well [14,15,16,17].

Strawberries are the most common fruits in the Mediterranean diet; they are rich in essential bioactive molecules, such as anthocyanins, flavonols, and flavanols, and tannins such as proanthocyanidins, ellagitannins, and gallotannins [18]. 

In the last years, several in vitro and in vivo studies have shown that among other plant-derived foods strawberries can be a precious ally in preventing and decreasing the risk to develop common diseases, including cancer; specifically, most of the intervention studies focused on strawberries have demonstrated promising effects on oxidative stress, inflammatory status, size or distribution of blood lipids, insulin response, and insulin sensitivity, while other studies have found no effects on these parameters [19].

They used a strawberry cultivar named Romina, released in 2011 from the breeding program of the Università Politecnica delle Marche, which is characterized by high adaptability to climatic conditions from the middle Adriatic sea to Central-Northern Europe, resistance to the main strawberry diseases, very early ripening, good taste with sweetness, and high firmness and shelf life [20]. The high sweetness of Romina fruit is highly appreciated by consumers and is recognized by the high content of soluble solids (SS) (7.7° Brix) combined with low total acidity (10.1 mEq NaOH/100 g) [20]. The high nutritional quality and health benefits of Romina fruit are also recognized for its good levels of vitamin C and folic acid, as well as for the high content of anthocyanins and antioxidant capacity [20]. In a recent study, we have evaluated the effect of the cultivar Romina on uterine leiomyoma and myometrial cells cultured in 2D, demonstrating its in vitro antifibrotic effect, induction of apoptosis, and inhibition of cell proliferation [21]. Therefore, our objective was to test the effect of this cultivar of strawberry on leiomyosarcoma cells. Cells cultured in 2D culture lack some essential aspects, such as cell–cell interactions and tumor architecture; thus, they fail to replicate the pathophysiology of cancer cells. The 3D systems guarantee some characteristics similar to those in vivo, including cell morphology, cell-matrix, cell–cell interactions, microenvironment, and distribution of oxygen and nutrients among others [22]. 

Therefore, the aim of this study was to test the in vitro effect of the Romina cultivar on leiomyosarcoma cells in vitro using agarose 3D spheroids. 

## 2. Material and Methods

### 2.1. Leiomyosarcoma Cell Lines Cultures

SK-LMS-1 cell lines were obtained from ATCC (Manassas, VA, USA). The cells were cultured in DMEM low glucose (Corning, New York, NY, USA) and added with 10% FBS (Euroclone, Milan, Italy) and 1% antibiotic P/S (Euroclone). The cells were incubated at 37 °C in 95% air and 5% CO_2_.

### 2.2. Spheroids in Agarose

For the formation of the spheroids, agarose gel was used. 

The agarose solution was made by dissolving 0.2 g of powder agarose (ThermoFischer, Carlsbad, CA, USA) in 20 mL of sterile distilled water; then, it was heated in the microwave for 1 min at around 85 °C and transferred to a 50 mL sterile falcon (Falcon, Corning).

Subsequently, cells previously detached from a T75 flask were inoculated with EDTA trypsin (Corning) and counted using the LUNA II automatic counter (Logos biosystem, Annandale, VA, USA) in the dissolved agarose gel and seeded in 6 wells (Falcon, Corning). In each well 1 mL of agarose + 1 mL of cell suspension (1 × 10^6^ number of cells) were plated by a serological sterile pipette (Corning). Then, when the agar solution containing the cell suspension completely solidified in each well, 1 mL of growth medium for leiomyosarcoma cells was added. Finally, the 6 wells were incubated with 5% CO_2_ at 37 °C.

To observe and count the spheroids, the images were acquired using the phase contrast optical microscope by Nikon (Nikon Europe, Amstelveen, The Netherlands) at times from 0 to 48 h.

### 2.3. Strawberry Fruit Extract and Cell Treatment

For the preparation of the strawberry extract, 50 g of fresh Romina fruits were added to 100 mL of a solution formed by methanol, Milli-Q water, and concentrated formic acid (in the ratio of 80:20:0.1), homogenized by using an Ultra-Turrax T25 homogenizer (Janke & Kunkel, IKA Labortechnik, Staufen, Germany), stirred at 22 g (ARE Magnetic stirrer, VELP Scientifica, Usmate, Italy) for 2 h at room temperature in the dark. After centrifugation at 2400× *g* for 15 min for two times, supernatants were filtered through a 0.45-µm Minisart filter (PBI International, Milan, Italy), concentrated, dried by using a rotary evaporator, and stored at −80 °C until cell treatments [13].

For the treatments of cells, leiomyosarcoma spheroids were washed with PBS (Corning) and then cultured in fresh low-glucose DMEM supplemented with 10% of FBS (Euroclone) and 1% P/S (Euroclone), and with 250 µg/mL of strawberry extract for 48 h. 

### 2.4. DAPI Staining

For DAPI staining, sterile PBS (Corning) was used in order to dilute the 4′,6-diamidino-2-phenylindole (DAPI) solution (ThermoFischer) to the final concentration of 300 nM. First of all, the spheroids were washed 1–3 times with sterile PBS and then were coated with the fluorescent dye solution of DAPI (300 nM) and incubated for 1–5 min, protected from light. 

Finally, the solution was removed by washing 2–3 times with PBS and images were acquired by using a Nikon fluorescent phase contrast light microscope.

### 2.5. Haematoxylin and Eosin

The spheroids were fixed with Paraformaldehyde 4% (Sigma-Aldrich, St. Louis, MO, USA) and embedded in paraffin. The paraffin sections were rehydrated by using both xylene and a descending-graded series of ethyl alcohol. After washing with the 50% alcohol, the sections were kept for five minutes in distilled water. Then, they were stained with haematoxylin (Bio-Optica, Milan, Italy) for 2 min, washed in distilled water, and then stained with Eosin (Bio-Optica) for 2 min; subsequently, they were washed in distilled water. Finally, they were dehydrated through an ascending graded series of ethyl alcohol and xylene and were mounted with Eukitt Solution (Orsatec GmbH, Kindler GmbH and Co., Bobingen, Germany).

### 2.6. Masson’s Trichrome Stain 

Masson’s trichrome stain was used to study the consistency, disposition, and amount of ECM [23]. This histochemical staining was performed using the kit from Bio Optica (Masson’s trichrome with green light) according to manufactory instructions. Briefly, the formalin-fixed and paraffin-embedded uterine leiomyosarcoma tissue sections were deparaffinized and rehydrated. The sections were washed in distilled water and postfixed with Bouin’s solution overnight. After this, the sections were subjected to the following steps: (i) rinsing with running tap water for 10 min; staining in Weigert’s iron haematoxylin working solution for 10 min, (ii) rinsing in running warm tap water for 10 min, (iii) washing in distilled water, (iv) counter-staining in Biebrich’s scarlet-acid fuchsine solution for 30 s, and, finally, (v) washing with distilled water. After differentiation in a phosphomolybdic–phosphotungstic solution for 15 min, the sections were covered with a light green solution and stained for 2–6 min; then, they were rinsed in distilled water and differentiated in a 1% acetic acid solution for 1 min. Following the washing in distilled water, the quick dehydration by using absolute alcohol and xylene and the mounting with a resinous mounting medium were performed. All the above reagents were purchased from Sigma-Aldrich (St. Louis, MO, USA).

### 2.7. RNA Extraction and Real-Time Polymerase Chain Reaction (PCR)

Total RNA was extracted by using Trizol reagent (Invitrogen, Waltham, MA, USA) following the manufacturer’s instructions. In order to digest the DNA, samples were treated with a ribonuclease-free deoxyribonuclease (Promega Corp., Madison, WI, USA), cleaned up and the RNA was concentrated with the ReliaPrepRNA Cell Miniprep System (Promega Corp.). The reverse transcription was performed by using the high-capacity cDNA reverse transcriptase kit (Applied Biosystems, Waltham, MA, USA) using 1 mg RNA. The real-time PCR was performed with the TaqMan method by using 100 ng cDNA in a final reaction volume of 10 mL. As a probe, the following TaqMan gene expression assays (Applied Biosystems) were used: (i) collagen 1A1 (Hs00164004_m1), (ii) fibronectin (Hs00365052_m1), (iii) versican (Hs00171642_m1), (iv) activin A (Hs00170103_m1), and (v) the housekeeping gene b-actin (ACTB) (Hs99999903_m1). The thermal cycle was programmed to perform initial denaturation at 95 °C for 20 s, followed by 40 cycles of 95 °C for 1 s and 60 °C for 20 s. The amplifications performed in the absences of reverse transcriptase enzyme were used as blank for each reaction.

### 2.8. Data Analysis

Statistical analyses were performed using GraphPad Prism version 9.5.0 for macOS (GraphPad). The data were analyzed using the *t*-test and Wilcoxon test; *p* < 0.05 was considered statistically significant.

## 3. Results

### 3.1. The Microscopic Observation of Spheroids

**Figure 1:**
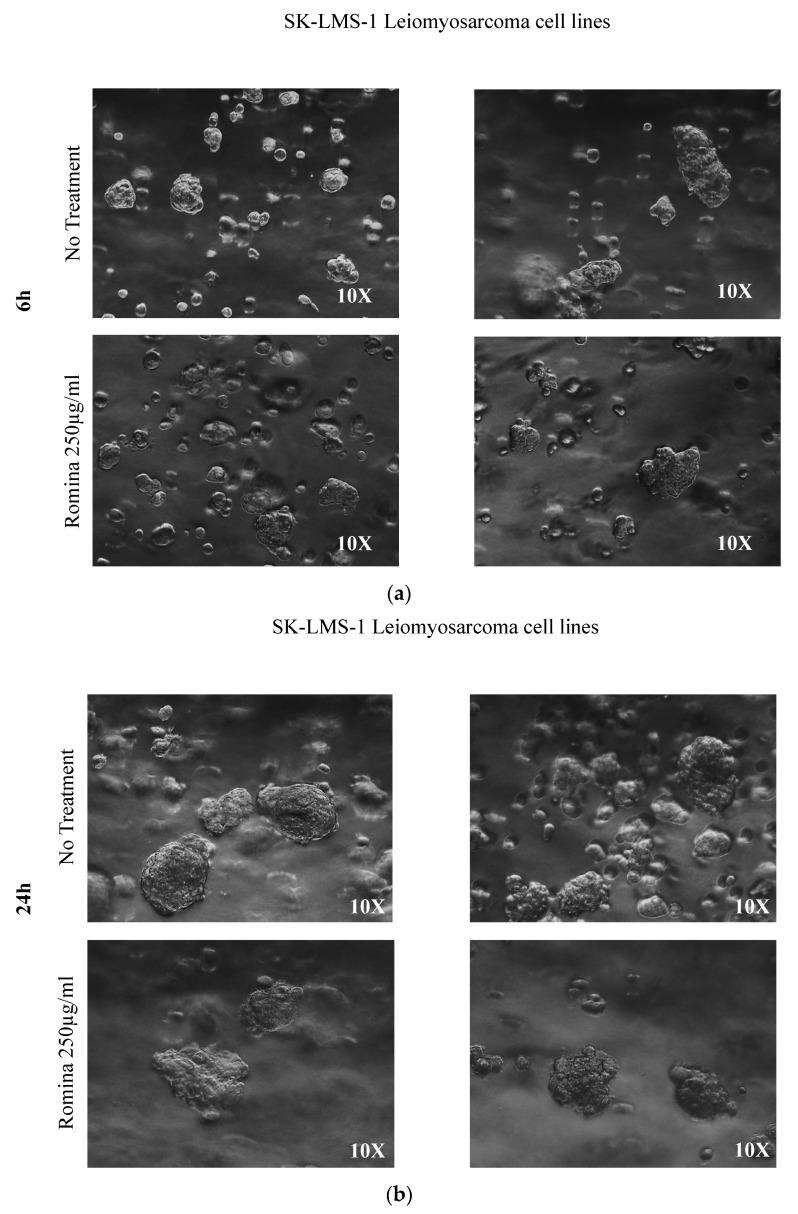

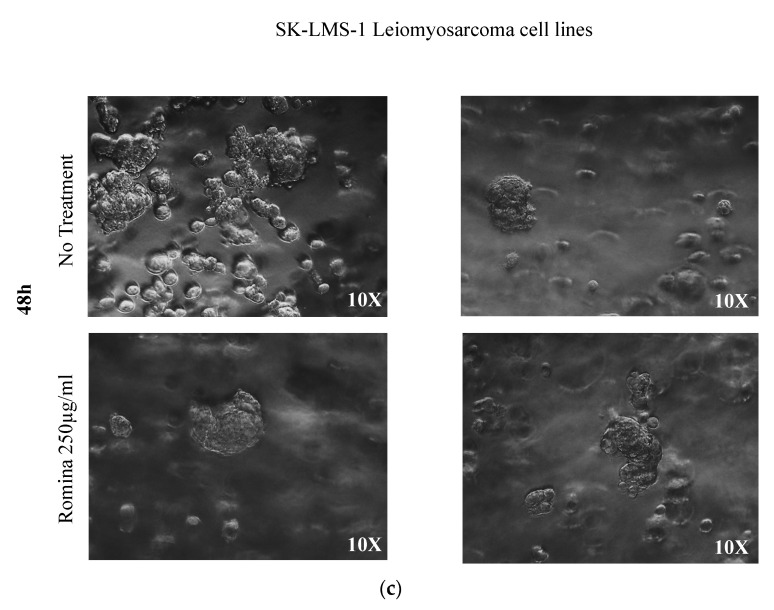
The representative images of the spheroid, made with a phase-contrast optical microscope. (**a**) Representative images of the spheroid after treatment of the cells in agarose with the cultivar Romina strawberry extract 250 µg/mL compared to the untreated control (6 h). (**b**) Representative images of the spheroid after treatment for 24 h of the cells in agarose with the cultivar Romina strawberry extract 250 µg/mL compared to the untreated control. (**c**) Representative images of the spheroid after treatment for 48 h of the cells in agarose with the cultivar Romina strawberry extract 250 µg/mL compared to the untreated control.

Pictures made with a phase-contrast optical microscope allowed the measurement of both the total number of spheroids and their area (Figure 1). The count showed that there was a significant reduction in the number of leiomyosarcoma spheroids after 48 h of treatments with the strawberry extract treatment (*p* = 0.0221) compared to the untreated control.

**Figure 2:**
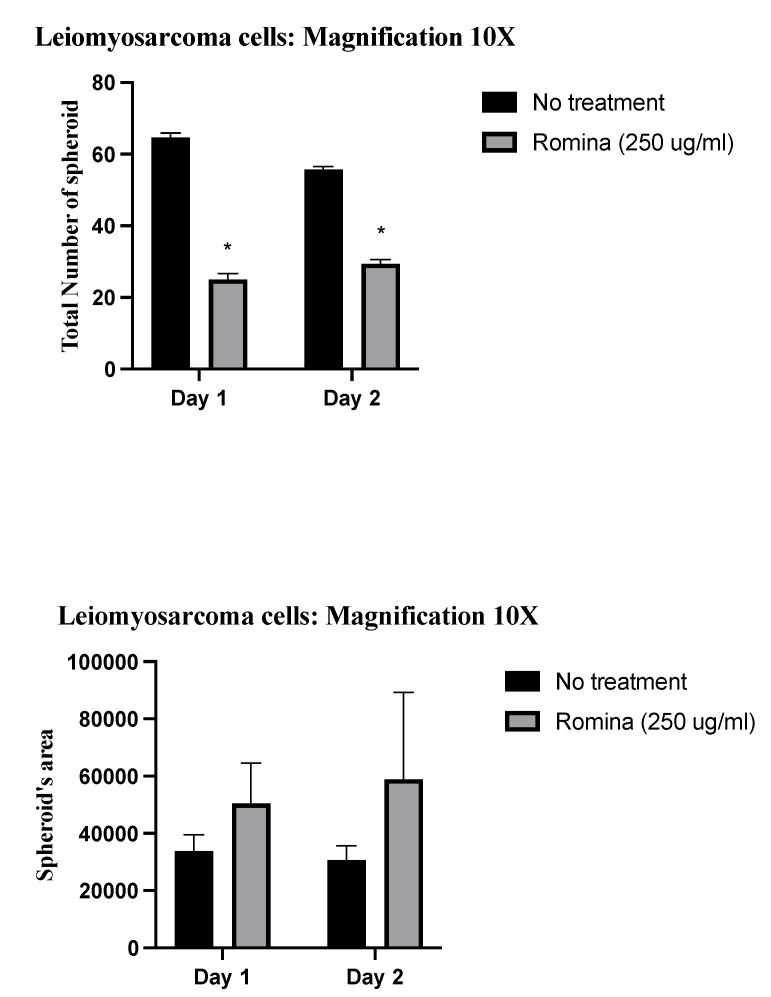
Schematic images of quantification. The images show the total number of spheroids, and in particular, show the significant decrease in total number after treatment with the cultivar Romina strawberry extract 250 µg/mL for 48 h compared to the untreated control (*p* = 0.0221). Furthermore, they show the total spheroid’s area increased of spheroid’s area after treatment with the cultivar Romina strawberry extract 250 µg/mL for 48 h compared to the untreated control. * means that is significant different.

In addition, the spheroids treated with strawberry extract tended to be bigger after treatment compared to the untreated control (Figure 1: qualitative pictures; Figure 2: quantification and measuring of spheroids).

In detail, representative images after 6 h, 24 h, and 48 h are presented in Figure 1, where it is possible to note that 6 and 24 h of treatment had no effect on the growth of the spheroids (Figure 1a,b); conversely after 48 h of incubation with strawberry extract a decrease in spheroid growth was noted compared to the untreated control (Figure 1c).

### 3.2. Morphological Characterization on Spheroid

**Figure 3:**
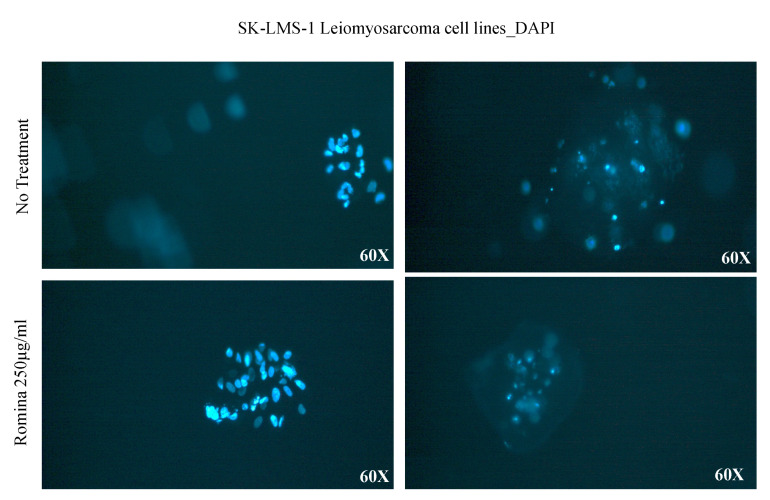
Representative images of a spheroid with DAPI staining. The images show spheroid formation after treatment with the cultivar Romina strawberry extract 250 µg/mL for 48 h compared to the untreated control.

In order to characterize the morphology of the spheroids, we performed the DAPI staining, haematoxylin and eosin, and Masson’s trichrome staining. The DAPI staining showed the aggregation of the cells in spheroid formation (Figure 3).

**Figure 4:**
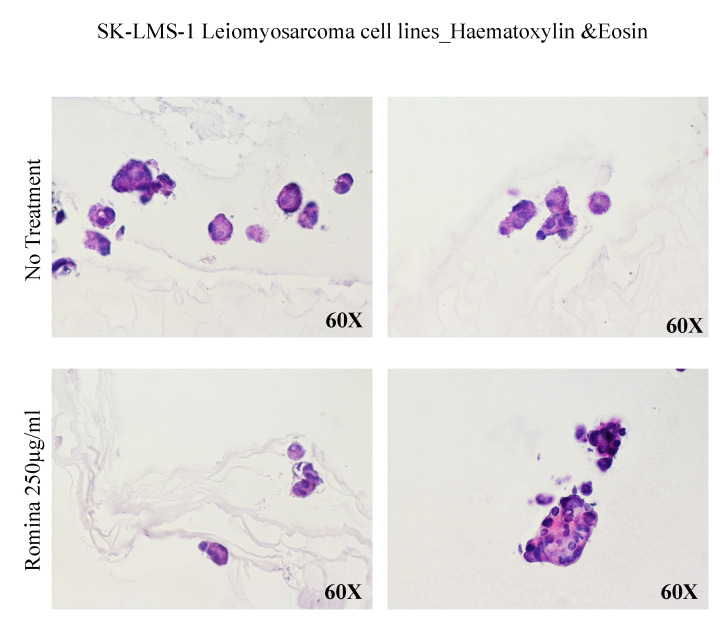
Representative images of a spheroid with haematoxylin and eosin. The images show the haematoxylin and eosin with spheroid after treatment with the cultivar Romina strawberry extract 250 µg/mL for 48 h compared to the untreated control.

**Figure 5:**
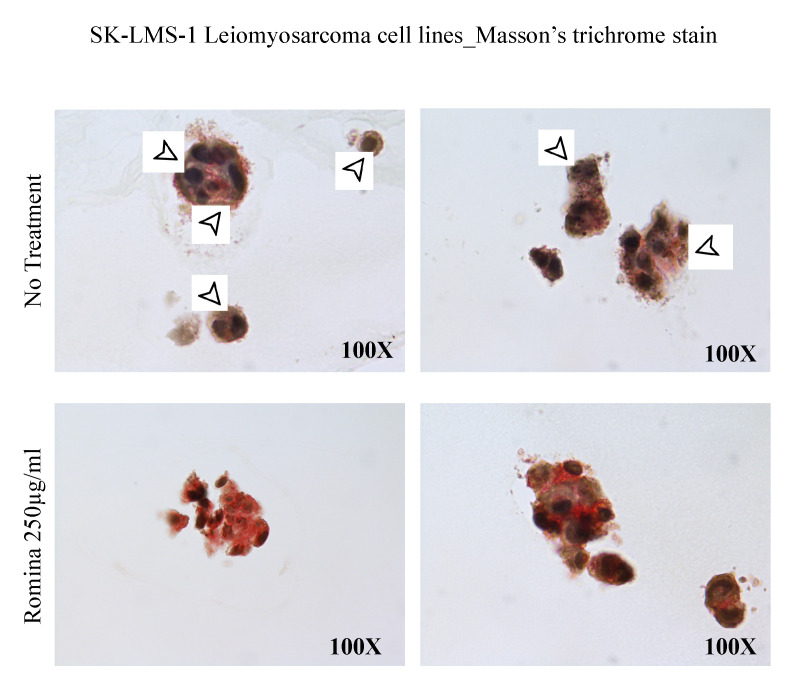
Representative images of a spheroid with Trichrome’s Masson. The images show the histochemical stain with spheroid after treatment with the cultivar Romina strawberry extract 250 µg/mL for 48 h compared to the untreated control. Arrows: collagen staining.

The spheroids were also observed after haematoxylin and eosin staining, showing an evident reduction of the number of spheroids treated with strawberry extract compared to the untreated control (Figure 4). In order to test the presence of an extracellular matrix (ECM), we performed Masson’s trichrome staining. This technique suggested a decrease in collagen formation compared to the untreated control; the collagen, stained in green, was present in the untreated control but resulted absent in spheroids incubated with strawberry extract (Figure 5).

### 3.3. The Effect of Cultivar Romina Strawberry Extract on Spheroid

**Figure 6:**
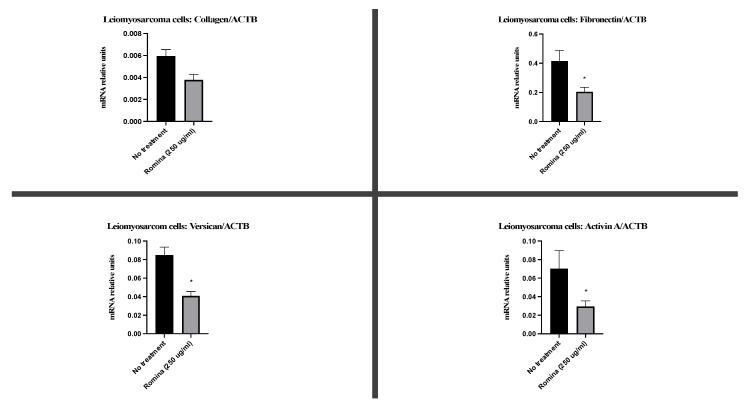
Real-time PCR on the spheroid. The representative images show the expression of ECM gene: collagen1a1, fibronectin, versiant, and activin A on spheroid treated with the cultivar Romina strawberry extract 250 µg/mL for 48 h compared to the untreated control. * means that is significant different.

Considering the above observations, we performed real-time PCR of ECM genes. Molecular analysis confirmed the morphological investigations in a significant and quantitative way. In detail, the treatment with strawberry extract decreased the expression of genes of the extracellular matrix, after 48 h of treatment. In fact, strawberry treatment slightly decreased the expression of collagen1a1 and significantly decreased the expression of fibronectin (*p* = 0.0312), versican (*p* = 0.0312), and activin A (*p* = 0.0312) in the spheroids of leiomyosarcoma (Figure 6).

## 4. Discussion

In the present study, we explored the effect of treatment with Romina cultivar strawberry extract on leiomyosarcoma spheroids formed inside an agarose matrix.

The three-dimensional culture of cells in agarose represents an effective method to perform morphological studies, since this matrix has transparent compact consistency and, therefore, it is suitable for fluorescent studies as well as for traditional morphological applications such as fixation, paraffin embedding, slicing, and further staining.

We found a significant reduction in the number of leiomyosarcoma spheroids observed with a phase-contrast optical microscope after 48 h of treatment with strawberry treatment compared to the untreated control.

Interestingly, the observation of Masson’s trichrome-stained slides suggested a reduced presence of ECM in spheroids treated with strawberries. The molecular investigations on the genes of the ECM confirmed that there was a trend of reduction of collagen, even if not significant, and a significant reduction of the expression of fibronectin, versican, and activin A in the presence of the strawberry extract.

In recent decades, there has been a growing interest in strawberries since their consumption could help prevent and reduce oxidative reactions that cause negative effects on human health, playing different roles in the development of chronic diseases and cancers [24]. In this study, we used the Romina cultivar due to its high content of antioxidants and bioactive compounds, such as polyphenols and vitamins, including Vitamin C. 

Vitamin C content is an essential molecule due to the large number of biological roles it plays in humans, reducing the incidence of cardiovascular and cerebrovascular diseases [25], various cancers [24], and other health problems such as lead toxicity [26].

Our research group over the years has developed several studies in vitro using phytochemicals on uterine leiomyoma cells, obtaining excellent results, and proposing the use of strawberries as a method of prevention and/or therapy [13,17,21].

The fact that strawberries seem to have a negative effect on the growth of leiomyosarcoma may open a new adjuvant therapeutic strategy for leiomyosarcoma. At the same time, those data reduce the doubts that could arise in the use of Romina strawberry cultivar in the therapeutic or preventive treatment of leiomyomas, even in case of misdiagnosis of leiomyosarcomas or in the case of STUMP.

Leiomyosarcoma is known to be the malignant counterpart of leiomyoma. While in leiomyoma it is well established that its growth is also due to an important increase in ECM proteins, in particular collagen 1A1, fibronectin, and versican, this is not so clear in leiomyosarcoma, although the role of ECM in soft-tissue sarcoma has been reported [27].

From this study, we obtained a trend of reduction after 48 h of treatment with Romina extract of collagen which is the central structural component of the ECM by maintaining cell morphology. It plays important roles in the regulation of proliferation, migration, differentiation, survival, and wound healing, as well as in fibrotic processes [28]. Various clinical trials are currently evaluating the therapeutic potential of targeting collagen to treat carcinomas [29]. It has been reported that leiomyosarcoma presents a particularly reduced fibrillar collagen content, and perhaps high collagen-matrix turnover [30]. However, the exact function of collagen in leiomyosarcoma still needs to be established and investigated.

Another important aspect that has emerged from this study is the significant reduction of fibronectin. Fibronectin is a glycoprotein of the ECM, which plays an important role in different processes, including cell adhesion, migration, growth, and differentiation, as well as fibrosis [27]. We have recently demonstrated that leiomyosarcoma cells express high levels of fibronectin [31]. This finding is extremely significant because fibronectin is also involved in tumorigenesis and metastasis. In fact, cell migration and invasion during tumorigenesis occur thanks to an adhesive interaction of cells with the ECM scaffold and proteolytic degradation on the ECM [32]. In cancer, fibronectin fibers direct the construction of the scaffold that will give rise to metastasis; its alignment, in fact, has a significant pathophysiological order, directing the invasion of tumor cells centrifugally away from the origin of the malignancy [33]. Cancer-associated fibroblasts (CAF) are one of the most abundant cell types in the tumor microenvironment with the ability to promote tumor growth; they exert their action by binding to ανβ3-integrin favoring the organization of fibronectin [34,35,36]. Under physiological conditions, fibroblasts can maintain homeostasis of the ECM under physiological conditions [37,38,39]. Conversely, CAFs secrete high levels of ECM proteins, such as fibronectin and type I and type II collagen, and express oncofetal isoforms of fibronectin [28,29,30]. Furthermore, CAFs can alter the architecture and physical properties of the ECM, affecting cell migration, invasion, and growth [40,41]. Indeed, CAFs generate tracks that cancer cells follow through force-mediated matrix remodeling and deformation of collagen I matrices [42,43]. 

The literature data reports fibronectin in different cancer tissues [24,25,26]; our previous study shows the presence of fibronectin in the central part of the leiomyosarcoma, suggesting that it may participate in leiomyosarcoma formation and progression by promoting tumor cell migration and invasion [34,35,36].

Our finding regarding the effect of Romina extract to reduce the expression of versican among the ECM is meaningful as well. In fact, this large chondroitin sulfate proteoglycan plays important roles in cell migration, cell adhesion, cell proliferation, tissue homeostasis, and inflammation [35]. The role of versican in leiomyosarcoma pathophysiology has been established since it has been demonstrated that SK-LMS cells produce a large amount of versican so that inhibiting its synthesis leads to a reduction of both proliferation and tumor formation when these cells are injected into nude mice [44,45].

No less important would seem to be the effect of strawberries on the reduction of the expression levels of activin A. This pleiotropic growth factor, belonging to the TGF-β superfamily, has an important physiological role in the reproductive system. It is classified as a hormone, growth factor, and cytokine, and has also important implications in fibrosis and tumor biology [46,47].

All these data encourage the investigation of the role of all ECMs further and deeper in leiomyosarcoma, as well as to further explore the effect that Romina cultivar strawberries may have since it seems promising.

In conclusion, our results demonstrated that strawberry extract from the Romina cultivar is able to reduce the leiomyosarcoma cell spheroids formation and has an antifibrotic effect. The data presented has been obtained in a three-dimensional culture that emulates the in vivo environment of leiomyosarcoma where also the microenvironment and fibrotic components are represented. In light of the reported results and of all the above considerations, we suppose that strawberry extract of the Romina cultivar could indeed have a role in the prevention and/or treatment of leiomyosarcoma or more generally in the control of the disease as an adjuvant to the surgical treatment which could remain fundamental. 

At present, it is, however, necessary to bear in mind the limitations of this study. In fact, even if 3D models represent a step forward for human research, the results obtained here should be validated in vivo on humans since, for instance, (i) the doses used in vitro are completely different from those used in vivo; (ii) in this in vitro study, all the aspects related to absorption and metabolism are totally lacking (and it is known that during digestion food metabolites are formed and exert the actual effects); and (iii) all the pharmacokinetic events have been not considered. 

## Data Availability

The data that support the findings of this study are available from the corresponding author upon reasonable request.
